# Polyclonal Intestinal Colonization with Extended-Spectrum Cephalosporin-Resistant Enterobacteriaceae upon Traveling to India

**DOI:** 10.3389/fmicb.2016.01069

**Published:** 2016-07-12

**Authors:** João Pires, Esther Kuenzli, Sara Kasraian, Regula Tinguely, Hansjakob Furrer, Markus Hilty, Christoph Hatz, Andrea Endimiani

**Affiliations:** ^1^Institute for Infectious Diseases, University of BernBern, Switzerland; ^2^Graduate School of Cellular and Biomedical Sciences, University of BernBern, Switzerland; ^3^Division for Infectious Diseases and Hospital Epidemiology, University Hospital BaselBasel, Switzerland; ^4^Swiss Tropical and Public Health InstituteBasel, Switzerland; ^5^Epidemiology, Biostatistics and Prevention Institute, University of ZurichZurich, Switzerland; ^6^Department of Infectious Diseases, Bern University Hospital, University of BernBern, Switzerland

**Keywords:** *E. coli*, travelers, food-chain, environment, pAmpC, ESBL, CTX-M, plasmid

## Abstract

We aimed to assess the intestinal colonization dynamics by multiple extended-spectrum cephalosporin-resistant Enterobacteriaceae (ESC-R-Ent) clones in Swiss travelers to India, a country with high prevalence of these multidrug-resistant pathogens. Fifteen healthy volunteers (HVs) colonized with ESC-R-Ent after traveling to India who provided stools before, after, and at 3- and 6-month follow-up are presented in this study. Stools were enriched in a LB broth containing 3 mg/L cefuroxime and plated in standard selective media (BLSE, ChromID ESBL, Supercarba) to detect carbapenem- and/or ESC-R-Ent. At least 5 Enterobacteriaceae colonies were analyzed for each stool provided. All strains underwent phenotypic tests (MICs in microdilution) and molecular typing to define *bla* genes (microarray, PCR/sequencing), clonality (MLST, rep-PCR), and plasmid content. While only three HVs were colonized before the trip, all participants had positive stools after returning, but the colonization rate decreased during the follow-up period (i.e., six HVs were still colonized at both 3 and 6 months). More importantly, polyclonal acquisition (median of 2 clones, range 1–5) was identified at return in all HVs. The majority of the *Escherichia coli* isolates belonged to phylogenetic groups A and B1 and to high diverse non-epidemic sequence types (STs); however, 15% of them belonged to clonal complex 10 and mainly possessed *bla*_CTX−M−15_ genes. F family plasmids were constantly found (~80%) in the recovered ESC-R-Ent. Our results indicate a possible polyclonal acquisition of the ESC-R-Ent via food-chain and/or through an environmental exposure. For some HVs, prolonged colonization in the follow-up period was observed due to clonal persistence or presence of the same plasmid replicon types in a new bacterial host. Travel medicine practitioners, clinicians, and clinical microbiologists who are facing the returning travelers and their samples for different reasons should be aware of this important phenomenon, so that better infection control measures, treatment strategies, and diagnostic tests can be adopted.

## Introduction

The human gut is increasingly recognized as an important reservoir of extended-spectrum cephalosporin-resistant Enterobacteriaceae (ESC-R-Ent), as increasing colonization rates by these pathogens are observed worldwide (Woerther et al., 2013). In particular, being colonized by ESBL-producing Enterobacteriaceae (ESBL-Ent) constitutes a risk factor to develop future extra-intestinal infections. Despite this fact, silent carriage of these multidrug-resistant (MDR) bacteria in the human gut is still poorly studied (Rogers et al., 2011; Van Der Bij and Pitout, 2012).

The highest colonization prevalence in the healthy population (~70%) has been reported in Southeast Asia (Woerther et al., 2013). Rates increased exponentially over the last decade and can be linked to several main factors: importation of high-risk clones (HiRC) via intercontinental travel, inappropriate use of antibiotics in different settings, high resistance rates in hospitals, poor hygienic measures (including food-handling), and high population density (Woerther et al., 2013). Therefore, it is not surprising that traveling to high prevalence countries constitute a risk factor to become colonized with these organisms (Van Der Bij and Pitout, 2012). Moreover, this importation of bacteria might facilitate the spread of MDR clones in low prevalence regions (Memish et al., 2003; Rogers et al., 2011). The problem has been highlighted in previous studies which reported individuals who acquired infections abroad with organisms encoding for new life-threatening resistance mechanisms (e.g., the first NDM-1 producer in a patient returning to Sweden or the importation of colistin-resistant MCR-1 producers from India), or which described resistance mechanisms in new bacterial hosts and/or genetic structures (Yong et al., 2009; Rogers et al., 2011; Seiffert et al., 2014; Bernasconi et al., 2016).

In recent years, an increased awareness for polymicrobial and/or polybacterial infections has also been achieved due to increased sensitivity of the microbiology diagnostic procedures (Short et al., 2014; Endimiani and Jacobs, 2016). However, we note that very few studies have identified multiple ESC-R-Ent colonizing the gut of healthy individuals (especially returning travelers) (Paltansing et al., 2013; Girlich et al., 2015; Stoesser et al., 2015), and very little attention has been attributed to this phenomenon.

In this work, we aimed to assess the colonization dynamics by multiple ESC-R-Ent clones acquired during a travel to India, a high prevalence country of ESC-R-Ent, to further understand the influence of this phenomenon in the shaping of the population structure of ESC-R-Ent in the human gut.

## Materials and methods

### Recruitment of volunteers and study design

Fifteen healthy volunteers (HVs) traveling to India were selected from a larger ongoing study to perform this longitudinal pilot analysis. These HVs were chosen because they: (*i*) provided stool samples before and after traveling (both within 1 week), (*ii*) were colonized with ESC-R-Ent after returning from their trip, and (*iii*) provided follow-up samples at 3 and 6 months after the trip.

All HVs were recruited at the travel clinic of the Swiss Tropical and Public Health Institute (Basel) where they signed an informed consent. Subjects were instructed to self-collect the native stools in sterile plastic containers and send them to the Institute for Infectious Diseases of the University of Bern (Switzerland). Ethical approval was obtained by the Ethikkommission Nordwest- und Zentralschweiz (EKNZ 239/12).

### Sample processing and isolate recovery

Stools (20 μg) were enriched overnight in 10 mL of Luria-Bertani (LB) broth containing a 30 μg cefuroxime disc. Enrichments were plated (50 μL) in BLSE, ChromID ESBL (bioMérieux), and a modified Supercarba plate containing imipenem to detect carbapenem- and/or ESC-R-Ent (Viau et al., 2016).

Species identification was obtained by using the MALDI-TOF MS (Bruker). At least 5 Enterobacteriaceae colonies per stool sample were analyzed. Strains were submitted to the double-disk synergism test (DDST) in cation-adjusted Mueller-Hinton plates (Becton Dickinson) supplemented with and without cloxacillin (250 mg/L; Sigma-Aldrich). Isolates yielding DDST results compatible with ESBL or plasmid-mediated AmpC (pAmpC) production were further characterized.

### Antimicrobial susceptibility testing (AST) and β-lactamase(s) characterization

ASTs were obtained using the microdilution Sentitre™ GNX2F plate (Trek Diagnostic Systems) and interpreted with EUCAST breakpoints, except for doxycycline and minocycline for which CLSI guidelines were used (CLSI, 2016; Eucast, 2016).

β-lactamase genes (*bla*) were identified using the CT103XL microarray (Check-Points). PCR/sequencing of the *bla*_ESBL_ and *bla*_pAmpC_ genes was carried out as previously done (Seiffert et al., 2013).

### Analysis of clonality and plasmid content

The repetitive extragenic palindromic PCR (rep-PCR) was implemented to assess clonal relatedness (Seiffert et al., 2013). Multilocus Sequence Typing (MLST) for *E. coli* (http://mlst.ucc.ie/mlst/dbs/Ecoli) and *K. pneumoniae* (http://bigsdb.web.pasteur.fr/klebsiella/klebsiella.html) were also performed. The phylogenetic group (PhG) for *E. coli* was also obtained (Escobar-Páramo et al., 2004).

Plasmids' incompatibility groups were assessed with the multiplex PCR based replicon typing (PBRT) kit (Diatheva).

## Results

### Demographic and clinical characteristics of travelers

As shown in Table 1, HVs were mostly females (73.3%) and middle-aged adults (average of 44 years). None of them was vegetarian or took anti-acids regularly. The average length of stay in India was 17.6 days (range 9–30 days). Four subjects reported having suffered from travelers' diarrhea. None of the HVs took antibiotics during their trip. During the 6-month follow-up period, several HVs traveled to other destinations (including India again).

**Table 1:** **Demographic and clinical data collected from the 15 analyzed healthy travelers (HVs) at different time points**.

**HV**	**Age/Sex**	**Job**	**VG**	**AC**	**Previous trips in the last 12 months**				**Trip in India**			**3 months after travel**			**6 months after travel**		
					**Countries**	**H**	**S/C**	**AB**	**Days**	**S/C**	**AB**	**S/C**	**AB**	**New Travel**	**S/C**	**AB**	**New Travel**
23	67/f	IT specialist	–	–	ITA (17 d); CRO (21 d); GER (2 d); MAL (7 d)	–	–	–	14	–	–	SvD, Na, AbC	Met	–	–	–	ITA (20 d)
26^a^	50/f	Speech therapist	–	–	ITA (28 d); FRA (7 d)	–	–	–	14	LD	–	–	–	ITA (5 d)	–	–	ITA (5 d)
29	31/m	Chemist	nd	nd	SPA (3 d); UK (3 d)	nd	nd	nd	15	–	–	–	–	PRT (2 d) FRA (3 d)	LD	–	DNK (6 d) FRA (6 d)
34	36/f	Art historian	–	–	UK (3 d); NED (4 d); ITA (2 d); AUT (2 d); DNK (5 d); GER (nd)	–	–	–	13	–	–	–	–	–	–	–	SPA (5 d)
37	43/f	Economist	–	–	ITA (nd); MOR (nd); JOR (nd)	–	–	–	27	–	–	–	–	LAO (14 d) VNM (14 d) KHM (14 d) MDG (14 d)	LD, AbC	–	PER and BOL (21 d)
41	68/f	Translator	–	–	–	–	–	–	15	–	–	–	–	–	–	–	–
43	37/f	Art historian	–	–	USA (14 d)	–	–	+, nd	17	LD, Na, AbC	–	–	–	–	–	–	UK (8 d)
50	33/m	Chef	–	–	AUT (5 d)	–	–	–	30	SvD, Na, AbC	–	–	–	–	–	–	–
56	42/m	Sales manager	–	–	ICE (14 d); BEL (5 d); UK (3 d)	–	–	–	9	–	–	–	–	–	–	–	ICE (10 d)
59^a^	50/f	Economist	–	–	ITA (14 d); CRO (21 d)	–	–	–	14	–	–	–	–	GER (5 d)	–	–	GER (6 d)
68	nd/m	Personal manager	–	–	IND (7 d)	–	–	–	20	–	–	LD	–	IND (17 d)	–	–	ITA (15 d)
80	26/f	Student	–	–	CRC (21 d); PRT (14 d); BEL (7 d)	–	–	–	19	–	–	–	–	–	–	–	FRA (5 d)
83	34/f	Jurist	–	–	GER (14 d); NED (7 d)	–	–	–	23	LD	–	–	–	–	–	–	–
97	42/f	Biologist	–	–	GRE (10 d); GER (nd)	–	–	–	20	–	–	–	–	FRA (10 d)	–	–	–
100	57/f	Librarian	–	–	–	–	–	–	14	–	–	–	–	–	–	–	–

*ID, identification code for the traveler; m, male; f, female; d, days; VG, vegetarian; AC, regular anti-acid intake; –, negative answer; nd, not defined; H, hospitalization; S/C, symptoms or complaints; AB, antibiotic intake; AUT, Austria, BEL, Belgium; BOL, Bolivia; CRC, Costa Rica; CRO, Croatia; DNK, Denmark; FRA, France; GER, Germany; GRE, Grece; ICE, Iceland; IND, India; ITA, Italy, JOR, Jordan; KHM, Cambodja; Jordan; LAO, Laos; MAL, Malta; MDG, Madagascar; MOR, Morocco; NED, Netherlands; PER, Peru; PRT, Portugal; SPA, Spain; UK, United Kingdom; USA, United States of America; VNM, Vietnam; LD, light diarrhea, SvD, severe diarrhea; Na, nausea; AbC, abdominal cramps; Met, metronidazole*.

a *Relatives traveling together*.

### Colonization timeline and individual ESC-R-Ent clones

Only three out of 15 HVs were colonized by ESC-R-Ent before traveling, whereas all of them had positive stools after return (Table 2). During the follow up period, six subjects were colonized at both 3 and 6 months.

**Table 2:** **Molecular features of the recovered individual clones detected during the study period among the healthy volunteers (HVs) traveling to India**.

**HV**	**Before traveling**		**After traveling**		**3 months after traveling**		**6 months after traveling**	
	**PhG-ST^a^**	**β-lactamase (Plasmid replicon types)^b^**	**PhG-ST^a^**	**β-lactamase (Plasmid replicon types)^b^**	**PhG-ST^a^**	**β-lactamase (Plasmid replicon types)^b^**	**PhG-ST^a^**	**β-lactamase (Plasmid replicon typing)^b^**
23	–	–	B_1_-ST200 (CC40)	DHA-1-like (FII)	–	–	B_1_-ST2521	CTX-M-1 (N)
26^c^	–	–	D_2_-ST3052 A_1_-ST3203 A_1_-ST685 A_0_-ST-New *K. pneumoniae*-ST48	CTX-M-15 (FII) CTX-M-15 (FII, B/O, K, I1) CTX-M-15 (FIB, HI1) CTX-M-15 (FIB) CTX-M-15 (FIIk)	–	–	–	–
29	D_2_-ST648 (CC648)	CTX-M-14 (FII, FIA, FIB)	D_2_-ST349	CTX-M-15 (X1)	D_2_-ST648 (CC648)	CTX-M-14 (FII, FIA, FIB)	–	–
34	–	–	B_1_-ST29 A_1_-ST-New	DHA-1-like (FII) SHV-12 (X1)	–	–	–	–
37	–	–	B_1_-ST4388 *Proteus mirabilis*	CTX-M-15+TEM-3-like (FII, FIB, P, I1) VEB-6 (not detected)	–	–	A_1_-ST48 (CC10) A_1_-ST744	CTX-M-24-like (I1) CTX-M-15 (FII)
41	–	–	A_0_-ST2325 B_1_-ST602 (CC446)	CTX-M-15 (FII, FIB) CTX-M-15 (FII)	–	–	D_2_-5841	CTX-M-1 (P, I1)
43	–	–	B2_2_-ST-New A_1_-ST617 (CC10)	DHA-1-like (FII, FIA, B/O) CTX-M-15 (FII, FIA, FIB, I1)	–	–	–	–
50	B2_3_-ST131 (CC131)	CTX-M-15 (FII, FIA, FIB)	B_1_-ST155 (CC155) A_1_-ST43 (CC10)	CTX-M-15 (FII, B/O, K) CTX-M-15 (I1, I2)	D_1_-ST394 (CC394)	CTX-M-15 (FII, FIB, B/O, K)	D_1_-ST394 (CC394)	CTX-M-15 (FII, FIB, B/O, K)
56	–	–	D_1_-ST-New	CTX-M-15 (FII)	D_1_-ST-New	CTX-M-15 (FII)	D_1_-ST-New	CTX-M-15 (FII)
59^c^	A_1_-ST652	CTX-M-15 (FII, B/O, K, I1)	A_1_-ST3203	CTX-M-15 (FII, B/O, K, I1)	–	–	–	–
68	–	–	A_1_-ST410 (CC23)	CTX-M-15 (FII, FIA, FIB)	A_1_-ST10 (CC10) A_1_-ST48 (CC10) A_1_-ST-New	CTX-M-15 (FII, HI2) CTX-M-15 (FII, HI2) CTX-M-15 (FII, Y)	–	–
80	–	–	A_1_-ST10 (CC10)	CTX-M-15 (FII)	A_1_-ST10 (CC10)	CTX-M-15 (FII)	–	–
83	–	–	A_1_-ST10 (CC10) A_0_-ST46 (CC46) D_1_-ST394 (CC394)	CTX-M-15 (I1, B/O, K) CTX-M-15 (I1) CTX-M-15 (FII, B/O, K, X1)	D_2_-ST648 (CC648)	CMY-42 (FII, FIA, FIB, I1)	D_2_-ST648 (CC648)	CMY-42 (FII, FIA, FIB, I1)
97	–	–	D_2_-ST2914 A_0_-ST841	CTX-M-15 (FII, I1) CTX-M-15 (FII, B/O, K)	–	–	–	–
100	–	–	B_1_-ST155 (CC155) B_1_-ST-New	CTX-M-15 (FII, FIB) CTX-M-15 (FII, FIB, B/O, K, I1)	–	–	–	–

*Note. “–”, negative for ESC-R-Ent*.

a *PhG, phylogenetic group; ST, sequence type. Clonal complex (CC) is shown within brackets. Phylogenetic group is shown only for E. coli clones. Species other than E. coli are specified. The new STs could not be assigned because whole-genome sequencing for these strains was not performed as requested by the curator of the E. coli MLST scheme (see Supplemental Table 1)*.

b *Only β-lactamases conferring resistance to extended-spectrum cephalosporins are shown*.

c *Volunteers traveling together*.

Among the 15 HVs, a total of 143 isolates yielded DDST phenotypes compatible with either ESBL or pAmpC production. rep-PCR analysis within each time point narrowed down the number of ESC-R-Ent isolates to 46 unique clones (data not shown), consisting of 44 *E. coli*, one *K. pneumoniae*, and one *Proteus mirabilis*. MLST analysis was performed in this sub-group of strains. Notably, ESC-R species other than *E. coli* were only recovered after traveling (Table 2).

### Antibiotic susceptibility of ESC-R-Ent

As shown in Table 3, none of the isolates detected in the post-travel stools were resistant to piperacillin/tazobactam or carbapenems. For non-β-lactams, higher rates of resistance (~35–40%) were observed for doxycycline, levofloxacin, ciprofloxacin, trimethoprim-sulfamethoxazole (STX), whereas resistance to minocycline and aminoglycosides was ≤ 20% and none of the isolates was resistant to tigecycline.

**Table 3:** **Distribution of β-lactamase types, antibiotic resistance rates and ***E. coli*** phylogenetic groups for the 15 volunteers**.

	**Overall period (*n* = 46, n° isolates, %)**	**Before Traveling (*n* = 3 n° isolates, %)**	**After Traveling (*n* = 28, n° isolates, %)**	**3 months after traveling (*n* = 8, n° isolates, %)**	**6 months after traveling (*n* = 7, n° isolates, %)**
**β-LACTAMASE TYPES**					
CTX-M-Group 1	36 (78.3)	2 (66.6)	23 (82.1)	6 (75)	5 (71.4)
CTX-M-15	34 (73.9)	2 (66.6)	23 (82.1)^a^	6 (75)	3 (42.9)
CTX-M-1	2 (4.3)	–	–	–	2 (28.6)
CTX-M-Group 4	3 (6.5)	1 (33.3)	–	1 (12.5)	1 (14.3)
CTX-M-14	2 (4.3)	1 (33.3)	–	1 (12.5)	–
CTX-M-24-like	1 (2.2)	–	–	–	1 (14.3)
SHV-12	1 (2.2)	–	1 (3.6)	–	–
VEB-6	1 (2.2)	–	1 (3.6)	–	–
DHA-1-like	3 (4.3)	–	3 (10.7)	–	–
CMY-42	2 (4.3)	–	–	1 (12.5)	1 (14.3)
**ANTIBIOTIC RESISTANCE**^b^					
Amikacin	1 (2.2)	0	1 (3.6)	0	0
Tobramycin	11 (23.9)	2 (66.7)	5 (17.9)	3 (37.5)	1 (14.3)
Gentamicin	6 (13)	2 (66.7)	3 (10.7)	1 (12.5)	0
Ciprofloxacin	19 (41.3)	3 (100)	10 (35.7)	3 (37.5)	3 (42.9)
Levofloxacin	23 (50.0)	3 (100)	11 (39.3)	5 (62.5)	4 (57.1)
Trimethoprim-Sulfamethoxazole	16 (34.8)	3 (100)	10 (35.7)	1 (12.5)	2 (28.6)
Doxycycline	21 (45.7)	1 (33.3)	11 (39.3)	5 (62.5)	4 (57.1)
Minocycline	10 (21.7)	1 (33.3)	6 (21.4)	2 (25)	1 (14.3)
Tigecycline	0	0	0	0	0
Colistin	1 (2.2)	0	1 (3.6)	0	0
Ticarcillin/clavulanate	10 (21.7)	2 (66.7)	4 (14.3)	2 (25)	2 (28.6)
Piperacillin/tazobactam	0	0	0	0	0
Cefotaxime	44 (95.7)	3 (100)	26 (92.9)	8 (100)	7 (100)
Ceftazidime	42 (91.3)	3 (100)	27 (96.4)	8 (100)	4 (57.1)
Cefepime	17 (37)	2 (66.7)	10 (35.7)	2 (25)	3 (42.9)
Aztreonam	42 (91.3)	3 (100)	24 (85.7)	8 (100)	7 (100)
Imipenem	0	0	0	0	0
Ertapenem	0	0	0	0	0
Meropenem	0	0	0	0	0
Doripenem	0	0	0	0	0
***E. coli*** **PHYLOGENETIC GROUPS**					
A	20 (45.5)	1 (33.3)	13 (50)	4 (50)	2 (28.6)
B1	8 (18.2)	0	7 (26.9)	0	1 (14.3)
D	14 (31.8)	1 (33.3)	5 (19.2)	4 (50)	4 (57.1)
B2	2 (4.5)	1 (33.3)	1 (3.8)	0	0

a *One isolates co-carries a TEM-3-like*.

b *Based on EUCAST breakpoints except for doxycycline and minocycline for which CLSI was used*.

During the follow up periods, the prevalence of resistance to fluoroquinolones and to minocycline increased, whereas for STX and aminoglycosides the resistance rates continuously decreased (Table 3).

### β-lactamase diversity over time

Pre-travel, the *E. coli* strains identified were CTX-M-15- and CTX-M-14 producers (Table 2). Of the 28 isolates recovered after traveling (26 *E. coli*, one *K. pneumoniae*, and one *P. mirabilis*), 89.3% were ESBL producers and the remaining 10.7% were pAmpC producers. CTX-M-15 was the most frequent ESBL identified (92.0%), followed by SHV-12 and VEB-6 (both 4%).

In the follow up period, a decreased in the diversity was observed. At 3 months, 75% of the strains produced CTX-M-15, while CTX-M-14 and CMY-42 were produced by 12.5% each. At 6 months, strains produced CTX-M-15 (42.9%), CTX-M-1(28.6%), CTX-M-24-like, and CMY-42 (14.3% each).

### Population structure of ESC-R-Ent

*E. coli* strains were highly diverse, with clones belonging to different STs and PhGs (Table 2). Before traveling, B2_3_-ST131, D_2_-ST648, and A_1_-ST652 strains were detected.

Among the 26 *E. coli* identified after returning from the trip, 22 (88%) yielded an individual ST. Nevertheless, 4 isolates (15%) belonged to the clonal complex (CC) 10. The PhG A was predominant (50%), followed by B1 and D (27 and 19%, respectively), being B2 PhG almost absent.

At 3 months, B2 or B1 PhGs were not identified, whereas PhGs A and D were equally represented. At 6 months, PhG D was the most frequent (57%), followed by A (29%) and B1 (14%) strains.

With regard to plasmids, those included in the F family were the most frequently identified (~80%), even though few other replicon types were observed (i.e., X1, I1, B/O, and K). F plasmids were also seen more frequently at 3 and 6 months, whereas other types identified after traveling were no longer recorded (Table 2).

### Shifts in molecular characteristics over time

Shifts in the molecular characteristics of the ESC-R-Ent recovered at the different time points were observed (Table 2). Three months after traveling, six HVs (HVs #26, 34, 43, 59, 97, and 100) were decolonized regardless of the number of clones identified immediately after return. For three HVs not colonized at 3 months (HVs #23, 37, 41), ESC-R-Ent were identified at 6 months; compared to each other, these strains carried a different β-lactamase type in a different ST and had a different plasmid content. For one subject (HV #29) colonized before traveling, the clone recovered just after the trip was different than the initial one; however, 3 months later the initial clone (D_2_-ST648-CTX-M-14) reappeared again (Supplemental Figure 1).

Persistent clones were also observed in HVs #56 and #80 as the same ST, plasmid content, and β-lactamase were identified at 3 and 6 months, respectively (Supplemental Figure 1). In the case of HV #83, there was a shift of clones and β-lactamases from the stools collected after traveling to those collected at 3 months; the same clone (D_2_-ST648) was identified after 6 months. Persistence of the β-lactamase in the same mobile genetic element (MGE) was suspected in the case of HV #50, since the same replicon types identified in the ST155 strain detected after traveling was identified in the ST394 detected at 3 and 6 months (Table 2, Supplemental Figure 1).

### Carriage of multiple ESC-R-Ent

After returning from India, 9 (60%) subjects carried more than one ESC-R-Ent, with a mean of 1.9 clones and a median of 2 clones (overall range of 1–5). Different *E. coli* STs were frequently identified colonizing the same individual harboring the same ESBL type. Nonetheless, carriage of different β-lactamases was also observed (HVs #34 and #43).

At 3 and 6 months, carriage of multiple clones was identified only in HV #68 and HV #37, respectively. In particular, HV #68 had three different CTX-M-15 *E. coli* producers at 3 months, whereas HV #37 had at 6 months two different STs harboring dissimilar CTX-M-types.

When different species were found together with *E. coli*, they could carry the same *bla* (i.e., CTX-M-15-producing *K. pneumoniae* for HV #26), or a different gene (i.e., VEB-6-producing *P. mirabilis* for HV #37).

## Discussion

In this study, we prospectively followed a group of 15 HVs who returned from India colonized at intestinal level with more than one ESC-R-Ent clone. We emphasize that only a few studies have explored the issue of the polyclonal colonization of travelers with these life-threatening Gram-negative pathogens (Paltansing et al., 2013; Girlich et al., 2015; Stoesser et al., 2015).

### Antibiotic susceptibility and β-lactamase diversity

The resistance profiles of the recovered ESC-R-Ent isolates (i.e., higher rates of resistance to fluoroquinolones, STX, and aminoglycosides) clearly emphasized the limited option available for antibiotic therapy in case of infection due to these pathogens. As expected, the most prevalent β-lactamase types identified in this study were the CTX-M-15 (Kennedy and Collignon, 2010; Tängdén et al., 2010; Peirano et al., 2011; Östholm-Balkhed et al., 2013; Paltansing et al., 2013; Kuenzli et al., 2014; Sole et al., 2014; Lübbert et al., 2015; Ruppé et al., 2015; Valverde et al., 2015; Barreto Miranda et al., 2016), whereas other β-lactamases (i.e., SHV-12-like, CMY-2-like, and DHA-type) were rarely detected (Östholm-Balkhed et al., 2013; Kuenzli et al., 2014; Sole et al., 2014). The frequent observation of F plasmids was also consistent with the great dispersion of these MGEs carrying bla_CTX−M−types_ among Enterobacteriaceae recovered from different settings worldwide (Carattoli, 2013; Mathers et al., 2015).

### Population structure shifts among ESC-R-Ent in returning travelers

Prior to travel, the HiRC B2_3_-ST131 and D_2_-ST648 were identified. These PhGs/STs have been found worldwide colonizing and/or causing infections in humans, both companion and food-producing animals, and wildlife (Woodford et al., 2011; Ewers et al., 2012, 2014; Pitout, 2012; Nakane et al., 2016). Interestingly, in the HVs analyzed these initial clones were replaced after the trip to India (Table 2).

Post-travel, the high heterogeneity of clones identified indicated numerous possible exposure sources with ESC-R-Ent. Given that isolates belonged mostly to A and B1 PhGs and to CC10, CC155, or CC23, we speculate that the acquisition of these clones could be linked to environmental exposure and/or the food-chain (Paltansing et al., 2013; Kuenzli et al., 2014; Valverde et al., 2015). Moreover, the fact that we found ESBL-Ent coding for the same β-lactamase enzymes, which are frequently reported in food-items and river waters, strengthens this hypothesis (Bajaj et al., 2015; Upadhyay et al., 2015; Zurfluh et al., 2015).

However, we should note that despite being unlikely, human-to-human transmission cannot be excluded, since people living in tropical or sub-tropical climates have PhG A *E. coli* strains as the predominant lineages in the gut (Escobar-Páramo et al., 2004; Tenaillon et al., 2010). One Indian study identified CTX-M-15-producing *E. coli* of PhG A as the most frequent ESBL-*E. coli* lineage colonizing the gut of healthy individuals (Escobar-Páramo et al., 2004; Dureja et al., 2014). Furthermore, the identification of A_1_-ST3203 in two HVs traveling together (#26 and #59), but having different pre-travel colonization status (Table 2), may suggest that the acquisition arises from a common exposure source or human-to-human transmission. Overall, the increasing recovery of PhG A and B1 *E. coli* is worrisome, due to their expansion in the clinical setting associated with the production of ESBLs (Woodford et al., 2011; Rodrigues et al., 2015).

In the stools collected at 3 and 6 months, several epidemiological shifts were observed. The persistence of the same clone was observed until 3 months for HV #80 and until 6 months for HV #56. Nevertheless, the identification of the same replicon types in a different ST, as observed for HV #50, suggests that *in vivo* conjugation may occur (van Schaik, 2015). However, we cannot exclude that the different clones identified during the follow-up can also be associated to new independent acquisitions, as several of these HVs have traveled to high prevalence countries during this period (HVs #23, 37, and 68; Table 1).

Among the different STs that could be consistently identified over time, ST648 and members of CC10 were the most frequent (Table 2). In addition, the majority of plasmids recovered belonged to the F family. This combination of clones and plasmids, which can easily adapt to the human host, could explain their persistence in the gut (Paltansing et al., 2013; Titelman et al., 2014). Intrinsic characteristics of these genetic backgrounds or factors conferring increased fitness (e.g., usage of alternative metabolic pathways), might also contribute to the persistent intestinal colonization (Alteri and Mobley, 2012).

### Colonization dynamics: Hypothetical pathways

According to our observations spanning a 6-month period, several pathways can be drawn when an initially non-colonized HV travels to a high prevalence setting: (*i*) clonal persistence after returning from the trip (e.g., HV #56); (*ii*) persistence of the MGE (e.g., HV #50); (*iii*) detection of a previously unidentified clone or independent new acquisition (e.g., HV #83), and (*iv*) clonal clearance (e.g., HV #26) from the intestinal tract after several months (Table 2).

In the case of previously colonized HVs, an additional pathway can be identified, as the initial clone can be still present after traveling, but below the detection limit and then re-emerges after some time (e.g., HV #29) (Paltansing et al., 2013). This phenomenon has been observed in patients colonized after an infection (Titelman et al., 2014).

### Polyclonality: Clinical and ecological implications

In our study, a median of 2 clones were identified in the stools of travelers after the trip, indicating that a polyclonal acquisition occurs when visiting a high ESC-R-Ent prevalence area. Interestingly, HV #68 returned to India in the follow-up period and then he was found colonized with three unique MDR clones, emphasizing the risk of polyclonal acquisition in this region. This overall phenomenon has been already perceived by other authors (Östholm-Balkhed et al., 2013; Paltansing et al., 2013; Lübbert et al., 2015; Ruppé et al., 2015; Valverde et al., 2015). However, only one study reported a mean of 1.8 clones among the travelers (being the polyclonal acquisition identified in 46% of them), but no bacterial population structure analysis was performed (Ruppé et al., 2015).

In our opinion, there are several important implications associated with the polyclonal acquisition of ESC-R-Ent. On the clinical level, physicians should be aware that extra-intestinal infections in patients who recently traveled in high ESC-R-Ent prevalence region might be due to MDR clones that are persisting in the gut. Therefore, the antibiotic treatment has to be tailored according to the ASTs results provided by the clinical laboratory and not empirically given (Kennedy and Collignon, 2010). At the diagnostic laboratory level, colony picking from the selective agar plates can lead to the identification of just one MDR isolate (e.g., identifying an ESBL-Ent ciprofloxacin susceptible, but not the ESBL-Ent resistant to the antibiotic), hindering the implementation of proper antibiotic treatment regiments (Kennedy and Collignon, 2010; Endimiani and Jacobs, 2016). At the colonization level, different pathways are possible. The genetic background of the bacterium could have a significant influence on its ability to persist in the gut. Moreover, *in vivo* conjugation can also propagate resistance mechanisms and/or novel adaptive characteristics into new bacterial hosts with stronger ability to persistently colonize the human gut (e.g., the HiRCs ST131 and ST648), further potentiating the overall spread of these MDR pathogens (Baquero et al., 2011; van Schaik, 2015). Moreover, it has also been demonstrated that bacteria with certain genetic backgrounds (ST131, ST648, and those of PhG A) do not suffer fitness upon acquisition of MDR plasmids. Therefore, particular combinations of very fitting STs with MDR plasmids might favor more successful colonization overtime (Johnson et al., 2015; Schaufler et al., 2016).

## Conclusions

A high polyclonal acquisition rate of ESC-R-Ent was observed when HVs traveled to India. These multiple clones can follow different pathways, ranging from clonal clearance to clonal and/or MGE persistence in the gut. The genetic backgrounds of the acquired clones suggest that this acquisition is linked to not yet well-defined non-human sources, with the food-chain and the environment being likely potential sources.

The effective implications of these overall findings are still not completely clear. However, it appears plausible that travel medicine practitioners, clinicians, and clinical microbiologists who are facing the returning travelers and their specimens for different clinical reasons should be aware of this important phenomenon, so that better infection control measures, treatment strategies, and diagnostic tests can be adopted. Studying the factors underlying ESC-R-Ent persistence in the gut is also extremely important to better devise decolonization and colonization prevention strategies in the near future.

## Author contributions

Conception and design (EK, CH, AE); acquisition of data (JP, EK, SK, RT); analysis and interpretation of data (JP, EK, HF, MH, CH, AE); drafting the work (JP, AE); revising it critically for important intellectual content (all authors); final approval of the version to be published (all authors); agreement to be accountable for all aspects of the work in ensuring that questions related to the accuracy or integrity of any part of the work are appropriately investigated and resolved (all authors).

## Funding

This work was supported in part by the Swiss National Science Foundation (SNF; grant number 153377 to AE).

### Conflict of interest statement

The authors declare that the research was conducted in the absence of any commercial or financial relationships that could be construed as a potential conflict of interest.
