# Supplementary material for: Polyclonal Intestinal Colonization with Extended-Spectrum Cephalosporin-Resistant Enterobacteriaceae upon Traveling to India
Source: Front Microbiol. 2016 Jul 12;7:1069. doi: 10.3389/fmicb.2016.01069 (PMC4940376; doi:10.3389/fmicb.2016.01069)
Supplement: Supplementary file 1 [file Presentation1.PDF]

Supplemental Figure 1. rep-PCR fingerprint of persistent colonization with the same clone

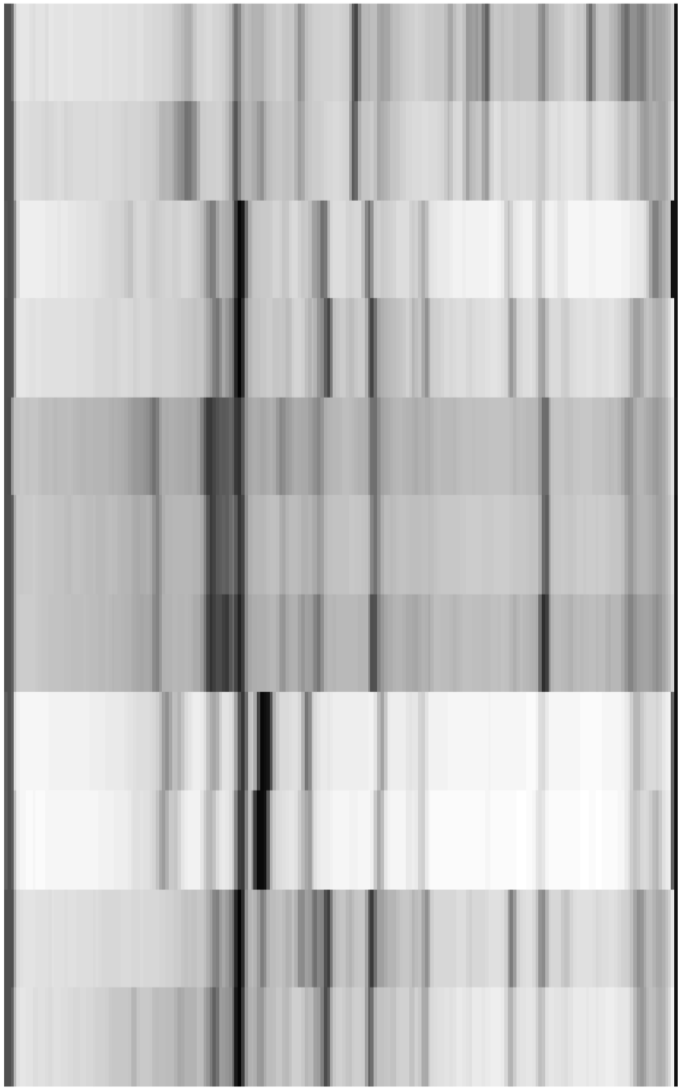

ID-Time Point [a]	ST	*bla*
80-M3	ST10	CTX-M-15
80-R	ST10	CTX-M-15
83-M6	ST648	CMY-42
83-M3	ST648	CMY-42
56-M6	ST-New [b]	CTX-M-15
56-M3	ST-New [b]	CTX-M-15
56-R	ST-New [b]	CTX-M-15
50-M6	ST394	CTX-M-15
50-M3	ST394	CTX-M-15
29-M3	ST648	CTX-M-14
29	ST648	CTX-M-14

[a] R - After Travel; M3 - 3 Months After Travel; M6 - 6 Months After Travel.

[b] Same allelic profile was observed for all MLST genes
