# Supplementary material for: Polyclonal Intestinal Colonization with Extended-Spectrum Cephalosporin-Resistant Enterobacteriaceae upon Traveling to India
Source: Front Microbiol. 2016 Jul 12;7:1069. doi: 10.3389/fmicb.2016.01069 (PMC4940376; doi:10.3389/fmicb.2016.01069)
Supplement: Supplementary file 2 [file Table1.PDF]

Table S1. Results of the MLST analysis for the *E. coli* strains with a new sequence type (ST)

Volunteer ID	Sampling time point	MLST scheme according to the University of Warwick							ST [a]	Best ST(s) match(es) (clonal complex, CC, when available)
		adk	***fumC***	***gyrB***	***icd***	***mdh***	***purA***	***recA***		
26	After trip	8	7	4	New	8	18	6	New	ST580, ST758, ST1124, ST1496, ST2325
34	After trip	10	11	4	1	8	8	11	New	ST5829, ST34 (CC10), ST98 (CC10), ST952, ST891
43	After trip	290	54	55	352	New	323	38	New	ST4134
56	After trip, 3 and 6 month follow-up	18	22	20	6	5	354	4	New	ST4692, ST130 (CC131), ST925, ST613 (CC31), ST5817
68	3 months after trip	New	7	4	8	12	8	2	New	ST159, ST746, ST813, ST2746, ST2919
100	After trip	43	41	New	18	11	8	6	New	ST1741

[a] The ST was not assigned because whole-genome sequencing for the strain was not performed as requested by the curator of the MLST scheme (http://mlst.warwick.ac.uk/mlst/dbs/Ecoli);
